# The Higher-Ed Organizational-Scholar Tension: How Scholarship Compatibility and the Alignment of Organizational and Faculty Skills, Values and Support Affects Scholar's Performance and Well-Being

**DOI:** 10.3389/fpsyg.2017.00450

**Published:** 2017-04-13

**Authors:** Milagros Pereyra-Rojas, Enrique Mu, James Gaskin, Tony Lingham

**Affiliations:** ^1^Latin American Studies Association (LASA)Pittsburgh, PA, USA; ^2^Business Administration, Carlow UniversityPittsburgh, PA, USA; ^3^Information Systems, Brigham Young UniversityProvo, UT, USA; ^4^Organization, Behavior and Leadership, Antioch UniversityYellow Springs, OH, USA

**Keywords:** scholarship identity, scholarship compatibility, scholarship productivity, academic alignment, well-being, job satisfaction, life satisfaction, person-organization fit

## Abstract

Scholars and institutions alike are concerned with academic productivity. Scholars not only further knowledge in their professional fields, they also bring visibility and prestige to themselves and their institutions, which in turn attracts research grants and more qualified faculty and graduate students. Many studies have been done on scholar productivity, and many of them focus on individual factors such as gender, marital status, and individual psychological characteristics. Also, a few studies are concerned about scholars' well-being. We propose a causal model that considers the compatibility of the scholarship dimensions valued by scholars and institutions and their academic alignment with actual institutional recognition and support. We test our causal model with data from a survey of 803 faculty participants. Our findings shed light on how the above academic factors affect not just academic productivity but also a scholar's well-being. Importantly, we show that academic alignment plays a crucial mediating role when predicting productivity and well-being. These results have important implications for university administrators who develop, and faculty who work under, policies designed to foster professional development and scholarship.

## Introduction

The dictum “publish or perish” has long been considered the battle cry for scholars in academia. Issues of promotion and tenure, as well as school rankings and credibility are largely determined by the extent of scholarship productivity. Scholarship productivity can be defined as the number and quality of academic publications. Many ways of measuring scholarship productivity have been proposed, including productivity indices such as the commonly used h-index^1^ (Hayes, 1983). In turn, scholarship productivity is linked to greater visibility, prestige, and access to financial resources (Hu and Gill, 2000). For these reasons, studying what factors contribute to, foster, or inhibit scholarship productivity is of paramount importance in academia.

This study differs from previous approaches by studying the role of academic-related values such as a scholar's scholarship identity, discussed in terms of the extent of preference given to specific Boyer's scholarship dimensions (Boyer, 1990; Van de Ven, 2007; Park and Braxton, 2013), institutional scholarship expectations (along same scholarship dimensions), the compatibility (or lack thereof) between the scholar and the institution with respect to these scholarship preferences—and the alignment with the values and skills actually recognized and supported by their institutions. We propose a model, based on a preliminary grounded-theory study (Mu and Pereyra-Rojas, 2015) and extant related literature (Braxton et al., 2002; Bland et al., 2005), to understand what role these factors play in scholars' academic productivity. We also explore how these academic factors affect a scholar's overall well-being—measured in terms of job and life satisfaction. This is important given the recent renewed focus in the literature on the importance of meaningful and satisfying work (Allan et al., 2016; Di Fabio and Kenny, 2016).

To explore these causal relationships, we conducted an initial pilot study with 212 faculty responses, and then a refined follow-up study with 803 faculty responses. Our findings show that scholarship compatibility—i.e., the compatibility of the intensity and breadth of the type of scholarship preferred by the faculty (scholarship identity) with respect to those expected by the institution—will drive academic alignment—i.e., alignment of the scholars' values and skills with actual institutional support and recognition—which in turn will lead to greater productivity and academic well-being.

This research has important implications for Higher-Ed organizations and the development of policies designed to foster faculty development and academic well-being. By determining the degree of compatibility between the institution and their facultys' scholarship interests, administrators may take action to either re-focus their scholarship priorities to be more compatible with faculty interests or simply hire faculty whose scholarly interests may be more compatible with those of the institution. This study is also important for faculty because it facilitates self-assessment of their own scholarship preferences and values and the degree of compatibility with the values of their institution.

This study is also important for the field of organizational psychology at large. In effect, a stream of research with a long tradition is constituted by person-organization (PO) fit which is loosely defined as the compatibility between people and organizations (Kristof-Brown, 2007). While different conceptualizations have been provided (Bretz and Judge, 1994), the most common and relevant for our study is the one provided by Chatman (1989) who discusses organizational fit in terms of the congruence of values between the individual and the organization. In this study, scholarship compatibility, defined as the congruence of scholarship valued by faculty with respect to those valued by the institution, constitutes an instantiation of a specific PO-fit value (scholarship). Our approach suggests that one of the ways to deal with the measurement challenges indicated by Kristof-Brown et al. (2005) and Morley (2007) is constituted by identifying a key value (in our case scholarship type) in the organization, its possible dimensions, and measuring the PO-value compatibility as will be shown in this study.

Next, we will discuss extant literature on the topic, develop our hypotheses, detail the research design and analysis, and discuss the implications of our current study in more detail.

## Literature review

For the purpose of this study, a literature search over a 10-year period was conducted using relevant keywords^2^ related to this study and producing 70 relevant papers, which were used to build the knowledge base for this study. We next discuss some of the major variables and findings from this literature as they relate to this study.

### Individual variables

Several studies have explored individual variables that may impact scholarship productivity (Bland et al., 2005; Garcia Cepero, 2007).

*Gender* as a predictor of scholarship productivity has been widely explored and most studies suggest that female faculty publish proportionally less than male faculty although some studies have not found differences (Cole and Zuckerman, 1991; Sax et al., 2002).

*Academic Rank* has, unsurprisingly, a positive significant effect on research productivity; that is, the highest the academic rank the more productive faculty is. This may be a reciprocal interaction since higher productivity is usually required for a higher rank and the higher rank also brings more prestige and resources which in turn leads to more productivity (Creswell and Bean, 1996; Bland et al., 2005).

Other individual characteristics have been studied that may be related to scholarship productivity, such as autonomy and commitment, motivation, research skills, and content knowledge. However, critical individual predictors are constituted by the *intrinsic motivation* to conduct research (Bland et al., 2005) and the extent of the academic communication network held by the scholar (Bland and Schmitz, 1986; Bland et al., 2005).

### Institutional variables

Institutional variables such as department size, presence of a doctoral program, culture, and mentoring have also been widely studied.

*Department Size* has been positively associated with greater productivity (perhaps because there are more opportunities to engage in collaborative research and learn from more experienced faculty). In addition, the presence of a “star” researcher is beneficial to overall faculty productivity (Dundar and Lewis, 1998). *Doctoral Program* presence at the institution is also positively correlated with a higher number of publications (Hayes, 1983).

*Faculty Teaching Load*, measured as a total number of teaching hours, has been also widely studied. Common wisdom and previous studies indicate that teaching load has an adverse effect on scholarship productivity (Alghanim and Alhamali, 2011; Akl et al., 2012). This seems logical because the more time a faculty is required to teach, the less time will be left for research. While most studies seem to agree on this point, at least one study has found that this is true only when the teaching load exceeds 11 h per week (Hu and Gill, 2000). The important guideline here is that teaching in moderation (about 25% of the normal 40 h perweek) would have no detrimental effect on a faculty's scholarship productivity (and in some cases it may constitute a source of ideas).

*Sufficient work time* allocated to research also has been found to be positively correlated to higher scholarsly productivity by several studies (Bland and Schmitz, 1986; Teodorescu, 2000; Bland et al., 2005). While this may seem common sense, it is interesting that at least one study concluded that research time should not exceed 80% of all work time or fall below 10% (Knorr et al., 1979) as reported by Bland and Schmitz (1986) who suggests that somewher in the 40% range may be ideal. While the validity of these values may be dated now, what is interesting is the suggestion that some time off actual research work may be also important for faculty productivity.

### Academic values and their institutional alignemnt

Our research extends of the previous studies discussed above by focusing on the professional values of scholars and their relationship to those of their institutions. Though there is anecdotal evidence of stress and struggles from scholars in their attempts to reconcile their professional values (e.g., what they deem worthwhile as academics) with those of the institution, only a few studies have systematically looked into the diverse aspects of scholarship (Boyer, 1990) and emergent identity types (Ward, 2003; Van de Ven, 2007) and how the alignment (or misalignment) of scholarly values and expectations from the institution and the scholar may foster or inhibit scholarly productivity.

For instance, Braxton et al. (2002) studied the degree of institutionalization of Boyer's scholarship models and warned that “the dominant model of a scholar and his/her productivity fails to align with the mission of most colleges and universities” (p. 12). In other words, they suggested that there is a misalignment between Boyer's scholarships valued by the academic and those valued by the institution. Still, it is recognized that values shape behaviors (Rokeach, 1973).

Values underlie the objectives of each of the forms of scholarship (Park and Braxton, 2013), and the degree to which scholars are socialized (or not) into the institutional values will affect scholarly productivity and the scholar's ability to obtain promotions, tenure, and beyond (Tierney and Rhoads, 1994). Because of this, it is also interesting that to date, not much work has gone into the investigation of scholarship identity, academic expectations and the well-being of scholars.

The scarcity of studies in this vein created an opportunity to further study this stream of research. For this purpose, we had to revisit Boyer's (1990) types of scholarship and Van de Ven's (2007) work on Engaged Scholarship and identify other studies that supported this work (Braxton et al., 2002; Ward, 2003). In particular, engaged scholarship has become extremely important for the management academic community because it constitutes the balance between the theoretical and trade nature of the discipline, providing an answer to the relevance of management scholarship (Kenworthy-U'ren, 2005; Peng and Dess, 2010). Based on this literature, five types of scholarship were considered for our study: Discovery, Teaching, Application, Integration, and Engagement. Since these scholarship dimensions are fundamental for this study, we next briefly discuss each.

### Five scholarship identities

#### Discovery

The scholarship of discovery could be defined as the art of creating knowledge. The discovery of this new knowledge “is absolutely crucial in our complex and vulnerable world” (Boyer, 1990: 18). However, it is not enough to create new knowledge; it must also be placed within the context of existing knowledge. In other words, it must be integrated within and across disciplines which leads to the next scholarship dimension.

#### Integration

The scholarship of integration underscores the need to give meaning to isolated facts, putting them in perspective (Boyer, 1990). Integration means making connections across disciplines, placing specialties in a larger context, in order to illuminate data in an enlightening way (Boyer, 1990). Still, understanding new knowledge in context is not enough as this new knowledge must be applicable.

#### Application

Boyer (1990) defines the scholarship of application as a dynamic process. In other words, this dimension of scholarship should not be interpreted as a one-way street in which discovered knowledge is simply applied. “New intellectual understandings can arise out of the very act of application” (Boyer, 1990: 23), and this type of dynamic interaction revitalizes theory and practice. In addition, knowledge must be communicated effectively to others, that is, it must be effectively taught.

#### Teaching

The scholarship of teaching lies not so much in the effective transmission of knowledge, but more in transforming and extending it through the development of creative and original ways of teaching (Boyer, 1990). Mu and Pereyra-Rojas (2015) found that many scholars who defined teaching as their main professional purpose would constantly investigate different ways of improving their teaching, effectively engaging in the scholarship of teaching.

#### Engaged scholarship

Engaged scholarship refers to the relationship between the expertise and resources of the university and the systems of community to address social, ethical, and civic problems (Van de Ven, 2007). The idea is to advance university-community partnerships in a collaborative effort in order to solve problems (Kennedy, 2003). As Boyer puts it, universities and colleges remain the greatest sources of hope for intellectual and civic progress; therefore, the academy must become a more vigorous partner in the search for answers to our most pressing social, civic, moral, and economic problems.

In our description of these types of scholarship, we have emphasized that a singular focus on one form of scholarship is insufficient. A balanced focus on all forms of scholarship is important and necessary to meet the demands and expectations of scholarship in the twenty-first century. Boyer (1990) suggested that the different dimensions of scholarship are not exclusive but instead complement and interact with each other. Furthermore, Van de Ven (2007) expanded the engaged scholarship dimension, based on the scholar-stakeholder relationship, which when present, could encompass other relationships.

## Theoretical framework

Our theoretical framework is informed by a qualitative study (Mu and Pereyra-Rojas, 2015) that investigated the lived experiences of scholars in Latin America and the US. Mu and Pereyra-Rojas (2015) interviewed 30 scholars representing 26 academic institutions, and the results suggested that factors such as the type of scholarship the participants identified with (e.g., discovery, application, teaching), institutional expectations and the academic alignment of the scholar's expectations and values with those of the institution played a key role in scholar productivity and well-being. Due to the lack of empirical studies in this area of research, the findings from Mu and Pereyra-Rojas (2015) study, being rooted in the tradition of grounded theory and supported by additional literature, constituted an appropriate platform on which to build our research model for this empirical study. Next we discuss, based on the extant literature, the derived constructs to be used in this study.

### Scholarship productivity

Scholarship Productivity is defined as a combination of the number of publications and their citations generated by the scholar's work during a specified period of time. Several studies, highlighted above, have been developed to measure and explain scholarship productivity. The most common measurement methods involve computing the number of peer-reviewed publications, citations, or a combination of the two. With respect to measures that involve a combination of both, the most common are the h- and g-index, h being the most conservative; that is, providing the same or a lower value than g. For this reason, the h-index will be used in this study. The h-index attempts to measure the cumulative impact of a scholar's output by looking at the amount of citations her work has received in journal articles published in the ISI^3^ as well as in books, book chapters, dissertations, theses, working and conference papers, reports, and journal articles not included in ISI and publications in non-English languages (Harzing, 2011). It is only through publication in suitable academic outlets and the degree of citations, not the dimension of scholarship favored by the academic and institution, that it is possible to assign value (for the academic community) to the scholarly contribution (e.g., in the business discipline, Academy of Management Journal (AMJ) is a suitable outlet for discovery-oriented scholarly work while Academy of Management Learning and Education (AMLE) is suitable for teaching-oriented scholarship). Still, there is an ongoing discussion about whether publications are the only or main way in which academic productivity should be measured (Aguinis et al., 2010; Northcraft and Tenbrunsel, 2012). While we agree with Northcraft and Tenbrunsel (2012) that publications (or rather citations) constitute a narrow way to measure academic productivity, the development of more pluralist indicators of scholarly impact as those proposed by Aguinis (2014) is still in its infancy. Because of this, we have adopted the citation index as a productivity measure due to its objectivity, ease of use, and broad acceptance in the academic community. For this reason, the h-index will be used to measure the degree of scholarship productivity.

### Academic well-being

While productivity has been a traditional concern in the management literature (Katzell, 1975), a more recent trend in positive psychology has been concern for the well-being of the worker (Layard, 2009; Dolan et al., 2011). In this study, well-being is defined as the extent of job and life satisfaction perceived by the faculty member. A review of the vast literature on academic job satisfaction showed that workers want some degree of work autonomy, recognition from their supervisors and colleagues, time for leisure and family life and fair pay (Bozeman and Gauchan, 2011). A review of the life satisfaction literature in relation to productivity indicated that the two are highly correlated. The presence of positive feelings is related to both more productive and happier people as demonstrated by Harter et al. (2002) in a meta-analysis of relevant studies. Based on the above, this study used job and life satisfaction as variables that suggest the degree of academic well-being, consistent with Layard's proposal of general well-being (Layard, 2009).

### Scholarship identity

Scholars tend to place different value on the specific dimensions of scholarship noted above (Boyer, 1990; Van de Ven, 2007). This in itself is not surprising; however, what is evident from the previous qualitative study that informs this research, is that this different valuing of scholarship dimensions constitutes in itself a source of strong academic identity for the scholars and would guide their decisions and provide a sense of purpose and mission about what to research and where (or if) to publish (Mu and Pereyra-Rojas, 2015). For instance, some scholars see themselves as expert knowledge seekers (and strongly value scholarship of discovery for this reason), while others are more interested in having a practical impact with their academic work (and may strongly value scholarship of application). For some scholars, teaching and any related scholarship, takes time away from their research, while for others it is part of their vocational call. Also, one type of scholarship does not exclude the others (e.g., a scholar driven by discovery may also value other identities), but scholars strongly identify with and tend to value one (or more) form(s) of scholarships over others. Furthermore, this scholarship prioritization is intrinsically linked to the scholar's sense of academic identity as is evident in quotes from Mu and Pereyra-Rojas' (2015) study; such as “*…[Research] I think is my mission,”* or “*My role is…to help produce a change of attitude…to solve societal problems” [Applied and/or Engaged Scholarship]*, and “*[Teaching] is what I always wanted since I was a child…becoming a professor.”* In summary, the valuing of specific dimensions of scholarship and how intensely they are valued provides a strong sense of scholarship identity to the scholar. In this study, we define scholarship identity as the extent (depth and breadth) of the scholar's preference for (identification with) different forms of scholarship. Depth refers to the intensity with which a scholar values a specific form of scholarship, while breadth refers to the scholar's valuation of more than one form of scholarship. We use the term scholarship identity because, as previously indicated, a strong preference for a specific type(s) of scholarship provides a sense of mission to scholars (Mu and Pereyra-Rojas, 2015). The higher the degree of scholarship identity, the more intense and broad the academic sense of mission and scholarship interests. In principle, and based on psychological hedonism theory (Moore, 2013), it would be expected that faculty would be more inclined to pursue scholarship dimension(s) they prefer the most. Scholarship identity also is important because current literature on motivation and mission-driven behavior supports the idea that people with a higher and broader sense of purpose tend to be more productive and satisfied with their lives (Porras et al., 2007). While faculty may have more than one preference, it is likely they will identify more with certain scholarship(s) (e.g., teaching) than others (e.g., discovery; Mu and Pereyra-Rojas, 2015).

### Institutional expectations

Institutional expectations is defined in this study as the depth and breadth of scholarship requirements imposed by the academic institution for promotion and tenure and as understood and perceived by the scholar for all the different forms of scholarship. While institutions may expect research along several scholarship dimensions, most likely they will give more importance to certain dimensions (e.g., discovery) than others (e.g., teaching). Also, these scholarship expectations may be independent from the faculty personal preferences. However, it may be anticipated, based on rational theory (Arrow, 1987), that faculty would tend to engage on institutionally desired scholarship given that this dimension(s) is more likely to be supported and rewarded by the institution; although this choice(s) may not be what they would personally prefer or identify with.

### Scholarship compatibility

So far, we have discussed the scholarship dimensions valued/preferred by the faculty (scholarship identity) and the scholarship dimensions valued/preferred by the institution (institutional expectations). We define scholarship compatibility as the extent to which the importance assigned by the faculty and the institution to each of the scholarship dimensions are coincident (or divergent) with each other. While either, the scholar or the institution, may favor similar dimensions of scholarship, the importance given to each dimension may still be different. If they are not coincident, this means there are discrepancies in the importance given to each of the dimensions by each of the two parties. For example, an institution may value scholarship of discovery very highly while the faculty may value it to a lesser extent while giving more importance to scholarship of teaching. The extent of these discrepancies or gaps along each of the scholarship dimensions will determine the extent of scholarship compatibility. In this study, we posit that the extent of scholarship compatibility (or lack thereof) will play a critical role in the faculty obtaining support and recognition from the institution as we will discuss next.

### Academic alignment

To put the scholar and institution's scholarship preferences in action, it is important that the institution will actually recognize and support with proper resources the faculty scholarship and related skills and values. Compatible scholarship interests (identity) will cause an alignment of the resources and support of the faculty scholarly interests to produce sound academic outcomes. A proper alignment of resources, skills, and preferences between the faculty and the institution is needed for the resources to be used optimally. For this reason, we define academic alignment as the extent of congruence between the scholar's identity, his/her skills, and institutional support. Institutions must support a scholar's preferences in order to enable the scholar to translate scholarly work into tangible academic outcomes. If this academic alignment is strong there is a better opportunity to obtain optimal results. We could expect that greater scholarship compatibility should lead to a greater academic alignment (i.e., academic support) because it is easier for the institution to recognize and support what they also value. If this academic alignment is weak (e.g., trying to complete a scholarship project using sophisticated quantitative methods when the institution supports mainly qualitative approaches), it will be very difficult for the scholar to get aligned with institutional recognition and support. Additionally, faculty will likely be stressed and unhappy about this conflicting struggle due to the resulting cognitive dissonance (Festinger, 1957). Mu and Pereyra-Rojas (2015) also noticed this situation. Although interviewed scholars did not talk about academic alignment as such, multiple scholars expressed this tension: “*Everything I was doing about the history of women, I was doing it on the side because nobody was interested,”* or “*I was negotiated…there were institutional interests directly inherited in what we needed to work on…”* (Mu and Pereyra-Rojas, 2015).

This need for academic alignment between the institution and the individual has also been discussed in the knowledge management literature. Tosey and Smith (1999) have theorized, using a systems complexity lens, that optimal performance (in our case scholarly productivity) occurs when the institutional and individual expectations, values and capabilities are aligned to form a self-reinforcing system. Furthermore, matching of institutional expectations, valued tasks (e.g., scholarship dimensions), and available resources may generate positive emotional states in the worker (Waterman, 1993). From a well-being perspective, the presence of positive feelings in the individual is also related to more productive and happier workers as was demonstrated by Harter et al. (2002) in a meta-analysis of Gallup studies about the relationship of well-being in the workplace with business outcomes.

In summary, our review of the preliminary qualitative study and relevant literature suggests that scholarship identity [scholarship(s) the faculty identifies with], institutional expectations [scholarship(s) expected by the institution], scholarship compatibility (extent of compatibility between scholarship identity and institutional expectations), and academic alignment (alignment of values, skills, and support between faculty and the institution) are key factors in the study of scholarship productivity and academic well-being. Furthermore, the mediating presence of academic alignment is critical for a scholar's productivity and well-being.

## Hypotheses

We have previously stated that scholarship identity provides a sense of mission to the faculty. It does not matter if the scholar identifies herself with more than one dimension of scholarship; on the contrary, multiple identities enhance one's sense of a purposeful, meaningful existence, and should enhance self-esteem and positive affect (Dietz and Ritchey, 1996). Similarly, we could expect that deeper and broader institutional expectations along the different scholarship dimensions should produce greater opportunities to work upon, no matter how different their preferences could be among the various faculty members.

However, we posit here that what is more important is the extent of compatibility between scholarship identity and institutional expectations. Scholarship compatibility is needed for faculty to create the opportunity to obtain proper recognition and support by the institution (academic alignment). In other words, scholarship compatibility between the faculty and institution scholarship expectations will lead to scholarly projects more likely to be supported and recognized by the institution; that is, will cause greater academic alignment.

Tosey and Smith (1999) suggested that people reach their highest performance when there is an organizational alignment of values and skills possessed by the individual and those valued and supported by the institution, as well as an alignment of the resources provided by the institution and those needed by the individual to perform the desired tasks. Similar findings have been proposed by Waterman (1993) and Harter et al. (2002). This academic alignment is caused by the compatibility of scholarship interests between the faculty and the institution. Hence, we hypothesize that academic alignment (congruence of institutional values, recognition, and support) mediates the positive effect of scholarship compatibility (commonality of scholarship preferences) into actual academic outcomes.

*H1: Perceived Academic Alignment mediates the effect of scholarship compatibility on scholarship productivity (h-index)*.

Motivation literature suggests that people who works with a higher sense of purpose in their jobs tend to not only be more effective but also happier (Frankl, 2006; Porras et al., 2007). We argue here that for this sense of purpose to occur optimally, the faculty must perceive there is a coincidence of scholarship preferences (i.e., mission and values) between them and their institutions, otherwise they will fall into a cognitive dissonance trap that will be counterproductive (Festinger, 1957). Conversely, scholars with greater extent of scholarship compatibility should experience greater well-being since they would avoid this conflict of interests. However, any sense of purpose or mission will translate into actual academic outcomes only through proper academic alignment. We have already argued that greater scholarship compatibility will cause greater academic alignment which is needed to obtain satisfactory academic outcomes. Optimal well-being occurs when institutional demands match desired (by the faculty worker) meaningful tasks and available resources (Waterman, 1993); that is, when there is academic alignment. This allows the scholars to combine their scholarship preferences with the available institutional resources to carry out their desired tasks. Should the institution recognize and support the scholar's identity, this will result in actual academic outcomes. This does not only result in more productive, but also happier workers (Harter et al., 2002). Proper academic alignment (which implies strong institutional support) will facilitate converting both faculty and institution expectations into actual academic outcomes; leading not only to higher productivity but also greater job and life satisfaction [“*my institution values what I value,” “my institution allows me to fulfill my call in life”* (Mu and Pereyra-Rojas, 2015)]. However, if these institutional expectations are not academically aligned, it may translate into lower job and life satisfaction (Tosey and Smith, 1999; Harter et al., 2002). Based on this, we can argue that scholarship compatibility will not only translate into higher scholarly productivity but also into greater academic well-being through the presence of academic alignment.

*H2: Perceived Academic Alignment mediates the positive effect of scholarship compatibility on academic well-being (H2a: Job Satisfaction and H2b: Life Satisfaction)*.

## Research design and methods

### Overview and study context

**Figure 1:**
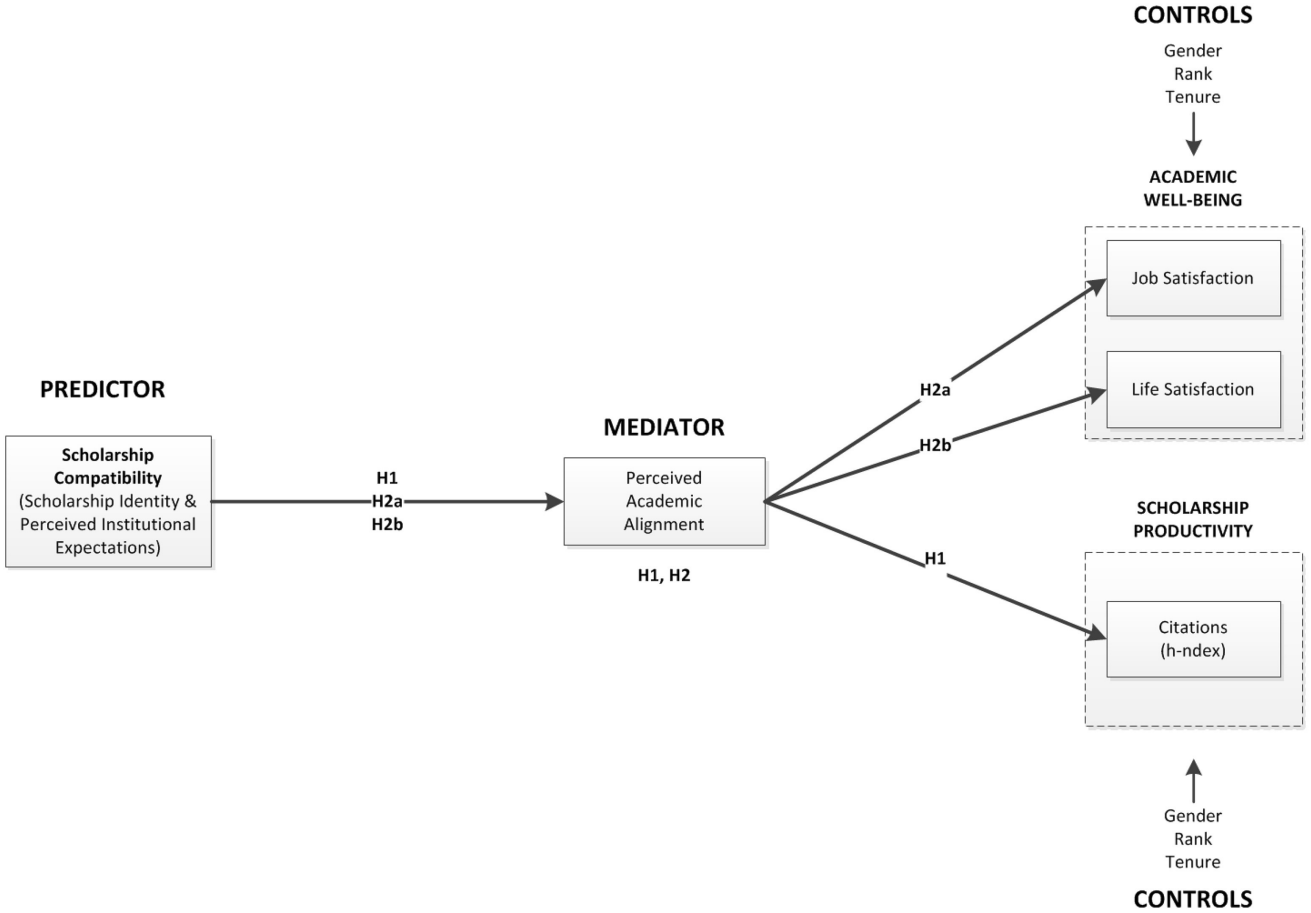
**Conceptual model with hypotheses**.

Testing our model (Figure 1) involved both the development of a reliable and valid scale followed by a pilot (preliminary study: scale development and pilot) and the ability to test its nomological validity (full study: data collection and hypothesis testing) by collecting data from different samples of the study population. In both phases, we used the survey method given that questionnaires are efficient for studies in which participants self-report to express their attitudes, beliefs, and feelings (Sheehan and McMillan, 1999; Teddlie and Tashakkori, 2009).

### Preliminary study: scale development and pilot

The reflective Likert scales for Scholarship Identity, Perceived Institutional Expectations and Academic Alignment were developed following DeVellis' (2003) scale development procedures. We first generated a large pool of candidates, based on the extant related literature (Boyer, 1990; Tosey and Smith, 1999; Van de Ven, 2007). Second, a group of five scholars (professors at two different universities) reviewed the pool of items for face and content validity. Third, a different group of eight experts (doctoral students) sorted the items into categories and obtained a high consistency rate of *r* = 0.90.

Finally, after refining the remaining 121 items, we explored their factor structure in a pilot test by surveying 588 scholars in three universities representing the three main university types as classified by the Carnegie Foundation for the Advancement of Teaching^4^ as well as in five major disciplinary categories^5^ in order to improve their clarity and accuracy. We received 212 responses during a 2-week period. Using permutated Exploratory Factor Analysis (EFA)^6^, items with low loadings (below 0.5) and high cross-loadings (when the difference between the two loadings was < 0.2) were eliminated. A eleven-subfactor structure (three composite factors) with 33 items for Scholarship Identity (5 sub-factors)^7^; 23 for Perceived Institutional Expectations (5 sub-factors) and seven items for Perceived Academic Alignment emerged and explained 69% percent of the total variance, supporting our conceptualization of three separate constructs.

The purpose of this study was to develop initial measures, test the survey and pilot the overall data collection process and analysis. Once the pilot showed the feasibility of the instrument and the study as such, the full study was performed.

### Full study: data collection and hypothesis testing

#### Sample and procedure

Hypotheses were tested in a stratified random sample of scholars to ensure representation by university category and discipline. Scholars from 12 universities in the United States representing, the three major university types and four discipline clusters were randomly selected for the study. The final instrument (web-based survey) was sent to 5,332 academics. The response rate was 23%. The percentage of fully completed surveys was 16% (of the original 5,332). 6% of the surveys were not used due to largely incomplete data, and the remaining 1% (with < 5% missing values) had those values imputed. The h-index was collected and matched by a doctoral student with access to the respondents' basic identifiers (first name, last name, and discipline) but not to the responses to maintain confidentiality. The final count of usable responses was 803 or 15% of the total number of scholars approached. Our response rate of 23% was well within the expected response rates for email surveys which vary from as low as 6% (Tse, 1995) to as high as 75% (Kiesler and Sproull, 1986). Table 1 reports the demographics for the 803 usable respondents.

**Table 1:** **Demographics**.

		**Humanities and social sciences**	**%**	**Natural and formal sciences**	**%**	**Applied sciences and professions**	**%**	**Total**	**%**
**University category**	Highest research	149	50	75	47	112	33	336	42
	Higher research	113	38	54	34	146	42	313	39
	Moderate research	37	12	31	19	86	25	154	19
	Total	299	100	160	100	344	100	803	100
**Tenure**	Yes	215	72	108	68	244	71	567	71
	No	69	23	42	26	76	22	187	23
	N/A	15	5	10	6	24	7	49	6
	Total	299	100	160	100	344	100	803	100
**Rank**	Lecturer	6	2	4	3	8	2	18	2
	Adjunct	2	1	1	1	4	1	7	1
	Assistant	57	19	33	21	60	17	150	19
	Associate	100	33	37	23	92	27	229	29
	Full	92	31	66	41	147	43	305	38
	University	12	4	6	4	6	2	24	3
	Emeritus	18	6	4	3	12	3	34	4
	Other	12	4	9	6	15	4	36	4
	Total	299	100	160	100	344	100	803	100
**Gender**	Male	156	52	114	71	177	51	447	56
	Female	143	48	46	29	167	49	356	44
	Total	299	100	160	100	344	100	803	100

#### Measures

All measures, except for h-index and Scholarship Compatibility were rated on a scale ranging from 1, “strongly disagree,” to 5, “strongly agree,” or 1, “very rarely,” to 5, “very frequently.”

##### Dependent variables

*h-index* This variable is an objective measure of academic productivity based on publications and citations in ISI academic outlets as well as in books, book chapters, dissertations, theses, working and conference papers, reports, journal articles not included in ISI and publication in non-English languages. This value is calculated using the “Scholar H Index Calculator” v. 2.3.6 (Ianni, 2012).

##### Academic job satisfaction

This is a perceptual measure referring to how content the scholar is with her academic job. This is one of two variables used to assess the overall academic well-being. Five-item scale was adapted from Brayfield and Rothe' Index of Job Satisfaction (Brayfield and Rothe, 1951).

##### Life satisfaction

This is a perceptual measure referring to how content the scholar is with her life in general. This is the second variable used to assess the overall academic well-being. Five-item scale was adapted from Diener's Satisfaction with Life Scale (Diener et al., 1985).

##### Predictor variables

All the measures for the independent variables were derived from Boyer (1990) and Van de Ven (2007) and complemented from Mu and Pereyra-Rojas' (2015) qualitative study.

##### Scholarship identity

This is a perceptual measure (17-item scale) referring to the extent (depth and breadth) of preference for a specific dimension(s) of scholarship along Boyer's (1990)^8^ four dimensions (sub-factors): discovery, integration, application, and teaching; as well as along one additional category: engaged scholarship by Van de Ven (2007). Items of each scholarship dimension were averaged to obtain a score for each of the dimensions. Therefore, scholarship identity is measured by a set of five numbers (range 1–5) that specify the extent of preference for each of the scholarship dimensions.

##### Institutional expectations

This is a 15-item perceptual scale referring to faculty member's perception of what their institution expects in terms of scholarship work. Perceived institutional expectation measures the University expectations in each of the five areas (sub-factors) of scholarship. Items of each scholarship dimension were averaged to obtain a score for each of the dimensions. Therefore, institutional expectations are measured by a set of five numbers (range 1–5) that specify the extent of preference for each of the scholarship dimensions.

##### Scholarship compatibility

We used a Compatibility Index (Garuti, 2012, 2016) to assess the degree of compatibility between scholarship identity and institutional expectations. Given that we have the intensity values along each of the five scholarship dimensions for both parties (scholar and institution) but we want to obtain a measure of the overall gap, we decided to treat scholarship identity and institutional expectations as two vectors with five coordinates each (each coordinate given by the importance chosen by either the scholar or the institution, respectively). In multi-criteria decision analysis (MCDA), it is common to compare the set of preferences of two decision-makers by means of plotting them as two vectors with normalized coordinates each and to calculate their degree of compatibility using the formula:

$$\left(1/2)\Sigma ({\text{a}}_{\text{i}}+{\text{b}}_{\text{i}})\;\times \text{ Min}({\text{a}}_{\text{i}},{\text{b}}_{\text{i}})\text{/Max}({\text{a}}_{\text{i}},{\text{b}}_{\text{i}}\right)$$

where the quotient Min (a_i_, b_i_)/Max (a_i_, b_i_) provides the Cos α-value and α is the angle between the two vectors. Also, i = 1,2…*N* for *N* coordinates (*N* = 5 in our case). In addition, a_*i*_ is the ith coordinate of the first vector (e.g., scholarship identity) and b_*i*_ is the ith coordinate of the second vector (e.g., institutional expectations).

When using the above formula, we can establish that there will be total compatibility between the two set of preferences when α = 0° (Cos α = 1). Geometrically, they correspond to two superimposed vectors with the same origin and end point. Total incompatibility will occur when the two vectors are perpendicular in the five-dimensional hyperplane (α = 90°, Cos α = 0). For this reason, the Compatibility Index provides a simple and objective way to assess the extent of compatibility between the scholarship preferences of the scholar with respect to those of the institution, ranging from 0 (incompatible) to 1 (fully compatible) along a continuous real number range^9^.

##### Mediator variable

*Academic alignment* Academic alignment refers to the scholar's perception that their scholarship identification (values) and skills are aligned with those valued and supported by their institutions. The extent of perceived academic alignment for each of the dimensions was measured using a 6-item perceptual scale developed by the authors for this study.

##### Control variables

We included three control variables to rule out explanations representing alternatives to our model (Figure 1). Based on our review of the extant literature we decided to control for the scholar's gender (male or female), rank (lecturer, adjunct, assistant, associate, full, university professor, and emeritus), and tenure (yes or no).

#### Data screening

No variables and no cases reported more than 5% missing data; thus we imputed data using the median where appropriate (Scheffer, 2002). All items were on a 5-point Likert scale (except for categorical variables), and for this reason no outliers were found or removed. We also checked standard deviations (as an indication of participant engagement), and our results showed that all items had standard deviations above 0.5, indicating that there was sufficient variance to proceed with all items (i.e., no respondents provided the same response for all survey questions; e.g., 3, 3, 3, 3, 3, 3…). All proposed relationships were tested for linear and curvilinear fit, and all effects were either significantly linear, or if not significant, were at least more linear (higher *F*-value) than they were curvilinear.

#### Exploratory factor analysis

An Exploratory Factor Analysis (EFA) was run during this phase to further refine the new scales (Scholarship Identity, Perceived Institutional Expectations, and Perceived Academic Alignment) using a randomly selected subset of the sample (*n* = 303). The Scholarship Identity Construct was reduced to 17 items which have factor loadings all larger than 0.53 (Discovery α = 0.879; Application α = 0.834; Teaching α = 0.842; Integration α = 0.886; Engagement α = 0.955). The Perceived Institutional Expectations construct was reduced to 15 items which have factor loadings larger than 0.50 (Discovery α = 0.697; Application α = 0.688; Teaching α = 0.824; Integration α = 0.909; Engagement α = 0.898). The Perceived Academic Alignment construct was reduced to six items which has a factor loading larger than 0.75 (α = 0.905). See Table 2—EFA Factor Loadings Table.

**Table 2:** **EFA factor loadings (standardized estimates)**.

**Item**	**CODE**	**Identity: Discovery**	**Identity: Application**	**Identity: Teaching**	**Identity: Integration**	**Identity: Engagement**	**Inst: Discovery**	**Inst: Application**	**Inst: Teaching**	**Inst: Integration**	**Inst: Engagement**	**Perceived Alignment**
1	IDDISC8	0.871										
2	IDDISC2	0.858										
3	IDDISC6	0.781										
4	IDDISC3	0.711										
5	IDAPP2		0.886									
6	IDAPP1		0.770									
7	IDAPP4		0.681									
8	IDAPP5		0.681									
9	IDTEACH7			0.856								
10	IDTEACH8			0.824								
11	IDTEACH9			0.735								
12	IDTEACH6			0.631								
13	IDINT6				0.898							
14	IDINT8				0.849							
15	IDINT3				0.783							
16	IDENG3					0.930						
17	IDENG4					0.902						
18	INSTDISC3						0.782					
19	INSTDISC2						0.687					
20	INSTAPP3							0.814				
21	INSTAPP5							0.730				
22	INSTAPP2							0.536				
23	INSTTEACH1								0.852			
24	INSTTEACH3								0.728			
25	INSTTEACH2								0.722			
26	INSTTEACH5								0.666			
27	INSTINT1									0.919		
28	INSTINT3									0.862		
29	INSTENG2										0.924	
30	INSTENG1										0.833	
31	INSTENG8										0.772	
32	INSTENG5										0.743	
33	ALIGN4											0.880
34	ALIGN6											0.808
35	ALIGN1											0.798
36	ALIGN2											0.761
37	ALIGN3											0.755
38	ALIGN8											0.712

#### Confirmatory factor analysis

Before testing the hypothesized model, we conducted a Confirmatory Factor Analysis (CFA) to confirm the factor structure of the self-reported variables using a subset of the sample consisting of the remaining responses not used for the EFA (*n* = 500). The results of the 5-factor model supported the hypothesized factor structure, as indicated by model fit metrics above recommended thresholds (Hu and Bentler, 1999; GFI = 0.879; NFI = 0.896; TLI = 0.950; CFI = 0.956; RMSEA = 0.036; PCLOSE = 1.000; SRMR = 0.0420).

To test for convergent validity we calculated the AVE. For all factors, the AVE was above 0.50 (Fornell and Larcker, 1981). To test for discriminant validity we compared the square root of the AVE to all inter-factor correlations. All factors demonstrated adequate discriminant validity because the square roots of the AVEs were greater than the correlations. We also computed the composite reliability for each factor. In all cases the CR was above the minimum threshold of 0.70, indicating that we have adequate reliability in our factors (Fornell and Larcker, 1981). See Table 3—Confirmatory Factor Analysis Loadings.

**Table 3:** **Confirmatory factor analysis loadings (standardized estimates)**.

**Item**	**CODE**	**Identity: Discovery**	**Identity: Application**	**Identity: Teaching**	**Identity: Integration**	**Identity: Engagement**	**Inst: Discovery**	**Inst: Application**
1	IDDISC8	0.853						
2	IDDISC2	0.841						
3	IDDISC6	0.800						
4	IDDISC3	0.736						
5	IDAPP2		0.861					
6	IDAPP1		0.828					
7	IDAPP5		0.717					
8	IDAPP4		0.689					
9	IDTEACH7			0.815				
10	IDTEACH8			0.797				
11	IDTEACH9			0.751				
12	IDTEACH6			0.683				
13	IDINT8				0.913			
14	IDINT6				0.852			
15	IDINT3				0.785			
16	IDENG3					0.961		
17	IDENG4					0.947		
18	INSTDISC2						0.999	
19	INSTDISC3						0.532	
20	INSTAPP3							0.804
21	INSTAPP5							0.735
22	INSTAPP2							0.611
**Item**	**CODE**	**Inst: Teaching**		**Inst: Integration**	**Inst: Engagement**	**Academic Alignment**	**Job Satisfaction**	**Life Satisfaction**
23	INSTTEACH1	0.883						
24	INSTTEACH2	0.779						
25	INSTTEACH3	0.723						
26	INSTTEACH5	0.697						
27	INSTINT3			0.940				
28	INSTINT1			0.897				
29	INSTENG2				0.941			
30	INSTENG1				0.929			
31	INSTENG8				0.778			
32	INSTENG5				0.568			
33	ALIGN4					0.906		
34	ALIGN1					0.811		
35	ALIGN6					0.751		
36	ALIGN2					0.740		
37	ALIGN3					0.738		
38	ALIGN8					0.690		
39	JOBSAT1						0.806	
40	JOBSAT5						0.738	
41	JOBSAT3						0.714	
42	JOBSAT4						0.665	
43	JOBSAT2						0.631	
44	LIFESAT1							0.904
45	LIFESAT2							0.893
46	LIFESAT3							0.827
47	LIFESAT4							0.749
48	LIFESAT5							0.621

#### Common method bias

Because some of the dependent variables (job and life satisfaction) were collected using the same instrument used to collect the independent variables, we conducted a common method bias test. The “common latent factor” method recommended by Podsakoff et al. (2003) for studies that do not explicitly measure a common factor (as in this study) was used to test for CMB. The difference between standardized regression weights with and without the common latent factor indicated that some of the items had differences >0.20. This suggests that CMB may be a problem. To adjust for CMB, we generated factor scores (for subsequent path analysis) while the CLF was present. Doing this creates factor scores that are adjusted for method bias. To ensure the CLF was not breaking our model by extracting too much variance (which may not always be strictly method variance), we reassessed CR and AVE with the CLF present. The results indicated that we still had reliable constructs (i.e., CR > 0.700 and AVE > 0.500).

#### Multivariate assumptions

Using the full model we tested for multi-collinearity using the Variable Inflation Factor for all of the exogenous variables simultaneously. The VIFs were all < 3.0, indicating that all of the exogenous variables are distinct predictors.

#### Analysis

Structural equation model was used to test the causal model. Composite variables were created in AMOS using the full sample (*n* = 803). To create composite factor scores, sub dimensions (Discovery, Application, Teaching, Integration, and Engagement) for each main factor (Scholarship Identity and Perceived Institutional Expectations) were aggregated.

The fitted structural model demonstrated good fit (GFI = 0.997; NFI = 0.996; TLI = 0.955; CFI = 0.997; RMSEA = 0.062; PCLOSE = 0.226; SRMR = 0.000). In order to achieve a good fit, we were required to co-vary errors terms for the dependent variables Job Satisfaction and Life Satisfaction as indicated by modification indices. Additionally, two regression path were added from the h-index to Job Satisfaction and life satisfaction showing significant results (standardized beta = 0.042 and 0.086, respectively). The actions we took allowed us to account for those potential correlations without having to explicitly theorize and test them^10^.

## Results

**Figure 2:**
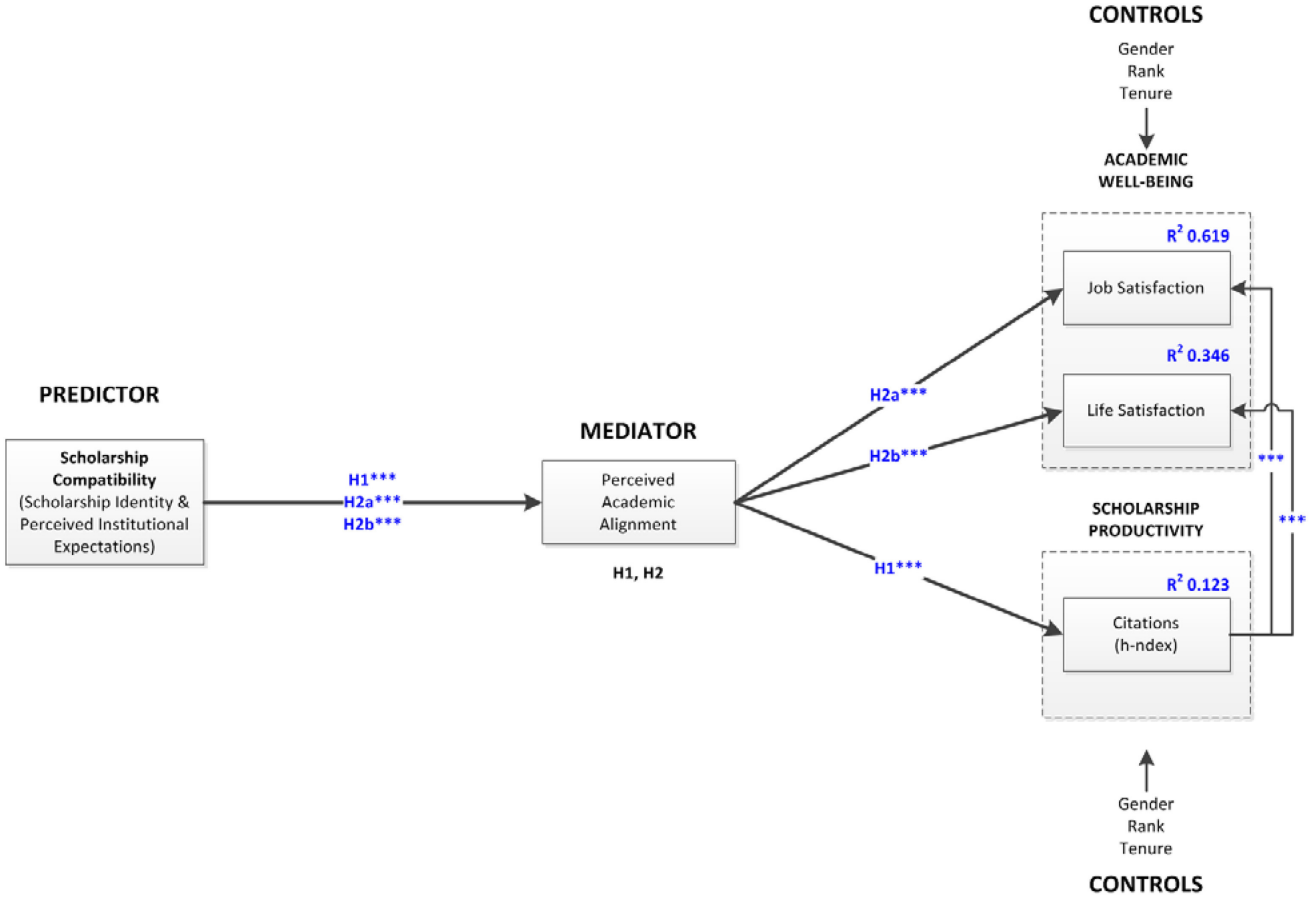
**Structural model with results**. ^***^p < 0.01; ^**^p < 0.05; ^*^p < 0.10. IE: Indirect Effect; NS: Not Significant. Solid lines (—) represent hypothesized significant paths; Dashed lines (*---*) represent hypothesized but not significant paths; Dotted lines (…) represent non-hypothesized significant direct paths.

Descriptive statistics of and correlations for the standardized variables of interest are shown in Table 4. Descriptive statistics for factor scores are presented in Table 5. A final structural model with significant hypotheses is presented in Figure 2.

**Table 4:** **Descriptive statistics and variable correlation matrix**.

**Item**	**Variable**	**Mean**	**Var**.	***SD***	**Min**.	**Max**.	**1**	**2**	**3**	**4**	**5**	**6**	**7**	**8**	**9**	**10**	**11**	**12**	**13**
1	Life Satisfaction	3.3574	0.486	0.697	0.840	4.570	**0.806**												
2	Identity: Discovery	3.7908	0.208	0.456	1.890	4.560	0.146	**0.809**											
3	Identity: Application	3.7414	0.321	0.567	1.380	4.730	0.082	0.131	**0.777**										
4	Identity: Teaching	4.3249	0.247	0.497	2.840	5.010	0.100	0.304	0.431	**0.763**									
5	Identity: Integration	4.3694	0.505	0.711	1.700	5.480	0.057	0.306	0.358	0.373	**0.852**								
6	Identity: Engagement	3.7370	0.776	0.881	1.210	5.670	0.032	0.004	0.633	0.328	0.406	**0.954**							
7	Inst. Expect: Discovery	4.5189	0.837	0.915	1.000	5.020	−0.007	0.055	−0.105	0.029	−0.001	−0.041	**0.800**						
8	Inst. Expect: Application	2.6388	0.397	0.630	1.070	4.860	0.051	−0.102	0.383	0.177	0.176	0.347	−0.080	**0.721**					
9	Inst. Expect: Teaching	3.7476	0.580	0.762	1.160	5.080	0.133	−0.014	0.194	0.164	0.058	0.158	−0.097	0.334	**0.774**				
10	Inst. Expect: Integration	2.6357	0.559	0.748	1.080	5.340	0.046	0.007	0.168	0.114	0.293	0.180	−0.032	0.530	0.254	**0.919**			
11	Inst. Expect: Engagement	2.5362	0.597	0.773	1.020	5.250	0.044	−0.113	0.345	0.146	0.251	0.475	−0.104	0.679	0.279	0.625	**0.818**		
12	Academic alignment	3.8511	0.670	0.819	0.970	5.450	0.558	0.235	0.072	0.129	0.014	−0.005	0.037	−0.016	0.217	0.045	0.006	**0.776**	
13	Academic job satisfaction	3.2026	0.235	0.485	1.170	4.070	0.786	0.275	0.158	0.181	0.092	0.037	0.007	0.092	0.289	0.128	0.106	0.710	**0.713**

*n = 803. Figures in bold in the diagonal are Cronbach's alpha*.

**Table 5:** **Descriptive statistics and construct correlation matrix**.

**Construct**	**Mean**	**Variance**	***SD***	**Min**.	**Max**.	**1**	**2**	**3**	**4**	**5**
1. Identity score	19.960	5.012	2.239	12.5	24.770	**0.958**				
2. Perceived institutional expectations score	16.077	5.231	2.287	7.69	24.120	0.248	**0.892**			
3. Academic job satisfaction	3.203	0.235	0.485	1.17	4.070	0.227	0.315	**0.852**		
4. Life satisfaction	3.357	0.486	0.697	0.84	4.570	0.122	0.200	0.814	**0.781**	
5. Perceived academic alignment	3.851	0.670	0.819	0.96	5.450	0.197	0.333	0.781	0.579	**0.713**

*n = 803. Boldface figures in the diagonal for composite scores (1–3) are Stratified Coefficient alpha*.

*Figures in the diagonal for composites (6–9) are Cronbach's alpha*.

### Mediation

Mediation was tested using 2000 bias corrected bootstrapping resamples in AMOS. The indirect effects (calculated as the product of the paths from the IV to Med and Med to DV) were analyzed for mediation. The results summarized in Table 6 provide support for our three mediation hypotheses.

**Table 6:** **Summary of hypotheses test results**.

**Mediation**	**Evidence**	**Supported?**
**H1**. Perceived academic alignment mediates the effect of scholarship compatibility on h-index	Indirect: 0.020^***^	**Yes**: Partial Mediation
**H2a**. Perceived academic alignment mediates the positive effect of scholarship compatibility on job satisfaction	Indirect: 0.107^***^	**Yes**: Partial Mediation
**H2b**. Perceived academic alignment mediates the positive effect of Scholarship compatibility on life satisfaction	Indirect: 0.075^***^	**Yes**: Full mediation
**Controls**	**Standardized beta**	
Gender → H index	−0.121^***^	
Gender → Job Satisfaction	−0.013 (ns)	
Gender → Life Satisfaction	0.015 (ns)	
Rank → H index	0.255 (ns)	
Rank → Job Satisfaction	0.033 (ns)	
Rank → Life Satisfaction	−0.008 (ns)	
Tenure → H index	−0.047 (ns)	
Tenure → Job Satisfaction	−0.031 (ns)	
Tenure → Life Satisfaction	−0.056^**^	
**Additional Paths**	**Standardized beta**	
H index → Job Satisfaction	0.042^*^	
H index → Life Satisfaction	0.086^***^	

Significant at

* p < 0.10,

** p < 0.05,

*** *p < 0.01*.

### Controls

Gender had a significant impact on h-index as well as Tenure on Life Satisfaction. Gender had a negative effect (standardized beta = −0.121^***^) on h-index. More specifically, females exhibited poorer performance. Tenure had a negative effect (standardized beta −0.056^**^) on Life Satisfaction. See Table 6 for a complete summary of hypotheses test results and Figure 2 for the final structural model with significant paths.

## Discussion

The results in Table 6 confirm our hypotheses; that is, they indicate that academic alignment significantly mediates the effect of scholarship compatibility on scholarship productivity (H1). With respect to academic well-being, academic alignment mediates the positive effect of scholarship compatibility on job satisfaction (H2a) and on life satisfaction (H2b). Discussed as a whole, these results provide valuable insight at theoretical and practical levels as will be discussed next.

### Theoretical contributions

This study makes several important contributions to the literature on scholarship productivity and academic well-being; as well as in the broader field of organizational psychology.

#### Contributions to scholarship productivity and academic well-being

First, the development of a measure to assess the extent of scholarship identity and institutional expectations along each of its dimensions and to be able to measure their extent of compatibility is an important contribution. To our knowledge, preferences for specific scholarship along Boyer and Van de Ven dimensions have only been discussed to date in terms of qualitative or ordinal preferences, but measures for a quantitative comparison of preferences have not been developed. Second, scholarship compatibility, mediated by academic alignment, has a significant positive effect on both scholarship productivity (measured as h-index) and academic well-being (job and life satisfaction). This means that institutions must carefully assess the scholarship expectations and the extent they are compatible with those of the faculty since this compatibility will be key to cause proper academic alignment that will eventually lead not only to greater scholarship productivity outcome but also to greater academic job and life satisfaction. Furthermore, the introduction of an objective measure (H-index), taken from multi-criteria decision analysis, to assess the extent of congruence between faculty and institutional expectations taking into account the five scholarship dimensions (discovery, application, integration, teaching, and engagement) is in itself a groundbreaking contribution to the academic literature. Finally, although not hypothesized, the structural model results (Figure 2) suggest that academic well-being occurs as a consequence of successful productivity. In other words, greater scholarship productivity may actually cause both greater job and life satisfaction.

#### Contributions to the broader field of organizational psychology: person-organization fit

As indicated in the introduction of our study, the person-organization (PO) fit construct, commonly defined as the congruency of values between the individual and the organization (Chatman, 1989), has a long tradition in organizational psychology (Kristof-Brown, 2007). Our study contributes to the theoretical advance of this research stream in a few ways.

First, one of the most recognized problems with the use of the PO construct has been the difficulty of measurement (Kristof, 1996) especially given different conceptualizations (Bretz and Judge, 1994). Our study proposes one simple approach to do this. Having identified the key value (scholarship type) to assess PO fit, we established the possible dimensions (Boyer's five types) for this variable and most importantly, we have developed a simple geometric vectorial approach (called the “compatibility index”) to compare the PO value fit—taking into account all the dimensions at once. This compatibility index approach could be also used for the same PO fit measurement purposes for different PO fit values as they may arise in different organizational contexts.

Second, our study provides empirical support that a greater degree of PO fit indeed leads to higher productivity and well-being, consistent with previous studies that suggest that PO fit has a positive (albeit moderate) effect on individual outcomes such as job satisfaction (Chen et al., 2016) and happiness (Moraes de Souza and Barreiros Porto, 2015), as well as with desirable organizational outcomes such as task performance and others (Verquer et al., 2003; Hoffman and Woehr, 2006).

Finally, Piasentin and Chapman (2006) have been very explicit that although there are different dimensions that represent distinct ways of perceiving PO fit, the dimensions have yet to be precisely defined or empirically tested. Our study suggests that the specific value and its dimensions need to be contextually defined and we also provide a novel way to measure this congruence (compatibility index), leading to results consistent with expectations based on the current state of the art of PO fit research studies.

### Practical implications

There are also practical implications of great importance for both scholars and academic institutions alike resulting from this study. First, scholars identify themselves with certain specific forms of scholarship and this provides a strong sense of scholarship identity and value—a sense of professional mission. Previously, this scholarship identification and its sense of purpose had not been made explicit; however, it is extremely important as can be seen in this study. By using the scholarship identity scale developed in the present study, scholars can understand the specific scholarship areas they value as well as have an overall measure of their scholarship identity. In other words, the scholarship identity scale allows scholars to explicitly and quantitatively assess their degree of scholarship identity. It is important to understand ourselves as scholars when making academic career and research decisions.

Second, similar to scholars, institutions value specific forms of scholarship differently. Some institutions may value scholarship of discovery above all else, while other institutions may be more accepting of broader forms of scholarship. Institutions rarely express their expectations for rank and tenure in terms of forms of scholarship but rather in terms of peer-review article requirements, class evaluations, etc. This study provides a scale for institutional expectations that allows a numerical assessment along each of the scholarship dimensions and as a whole. Furthermore, given the demonstrated impact of the compatibility between scholarship identity and institutional expectations on both academic productivity and well-being (mediated by academic alignment), institutional leaders could use our instruments to engage faculty into the design and/or improvement of institutional scholarship expectations. For example, an academic administrator could find it extremely useful to assess if the faculty expectations are consistent with the scholarship the institution is interested in promoting. Based on this, the administrator could decide to re-formulate their institutional expectations to ensure optimal results (productivity and well-being). Another alternative would be to reinforce the faculty body (e.g., new hires) in those scholarship areas that are not currently favored by the existing faculty^11^.

Third, scholars in general could find it useful to compare institutional expectations, in terms of scholarship dimension, with their own scholarship preferences (identity), in order to assess the degree of congruence (or incongruence) between the two perspectives. This could lead to conversations about expectations with the institutional leaders or even to a decision to move to a more compatible institution.

Fourth, administrators can also assess if there is a proper academic alignment of the faculty and institutional expectations along the required support and resources needed to produce academic outcomes. As shown in this study, academic alignment is important to mediate the effects of scholarship compatibility to obtain higher productivity and academic well-being.

In conclusion, by using our instrument, an administrator can detect serious problems in either the faculty identity composition and institutional alignment with the faculty scholarship preferences and support. Similarly, scholars can assess quantitatively if they are working in the proper institution aligned with their professional expectations. This study shows that both scholarship compatibility and academic alignment are key to lead to actual academic outcomes and well-being.

### Limitations and future research

To extend the model to academia worldwide we will need to survey faculty in other countries. A second limitation is the fact that we did not have enough responses needed to test differences among individual disciplines. It would also be convenient to move from a broader context of disciplines (i.e., natural and formal sciences, applied sciences and profession, and social science and humanities) to a more specific (e.g., management) to find out if the proposed concept and model requires discipline specific modifications.

Another broader question is whether the h-index should be the key measure of academic productivity or whether it should be combined with another measure. It also asks if the indices are enough to measure the different possible forms of scholarship (i.e., discovery, application, teaching, integration, and engagement) outcomes. Further studies may be needed to explore alternative ways to measure scholarly productivity, taking into account the scholarship forms discussed in this study. Also, due to similar reasons, it may be interesting to explore if the proposed model holds for each form of scholarship individually.

Finally, this consideration of congruence between professional values of the individual and the organization may be taken to a broader organizational context, beyond the context of higher-education institutions. Here, the question is if there are generic professional values applicable across industries or if specific PO variables would need to be developed according to each specific industry. Our study suggests that most likely, specific person-organization value(s) would need to be defined in each case; however, our compatibility index approach could still be used to effectively measure the degree of PO fit in each case.

## Ethics statement

Institutional Review Board at Case Western Reserve University. We followed all guidelines and procedures for using human subjects as dictated by the IRB at CWRU. Accordingly, we obtained exempt status after a review by the board. No one in the “at risk” group. Just scholars.

## Author contributions

MP designed and executed the study under the guidance of EM, JG, and TL. JG advised largely regarding the methodology and analyses while EM advised regarding framing and positioning. TL advised on the content and study formulation.

### Conflict of interest statement

The authors declare that the research was conducted in the absence of any commercial or financial relationships that could be construed as a potential conflict of interest.

## Footnotes

^1^The h-index uses Google Scholar listings which include citations to books, book chapters, dissertations, theses, working and conference papers, reports, and journal articles not included in ISI as well as publication in other languages.

^2^For example for the *academic alignment* construct the following keywords were used: academic alignment, values alignment, value alignment, organization alignment, organizational alignments, and employee alignment. In total 48 different keywords were used and the search took place using web of science.

^3^The Institute for Scientific Information (ISI) maintains citation databases covering thousands of academic journals, including a continuation of its longtime print-based indexing service the Science Citation Index (SCI), as well as the Social Sciences Citation Index (SSCI), and the Arts and Humanities Citation Index (AHCI). This database(s) allows a researcher to identify which articles have been cited most frequently, and who has cited them.

^4^R1, Highest research activity; R2, Higher research activity; R3, Moderate research activity The Carnegie Classification of Institutions of Higher Education (n.d.). Institutions were included in these categories if they awarded at least 20 research/scholarship doctorates in 2013–2014. Professional practice doctoral degrees (J.D., M.D., Pharm.D., Aud.D., DNP, etc.) were not counted for the purpose of this criterion.

^5^(1) Humanities: History, English, and Linguistics; (2) Social Sciences: Anthropology, Economics, Political Science, and Sociology; (3) Natural Sciences: Life Sciences and Physics; (4) Formal Sciences: Computer Science and Mathematics; and (5) Applied Sciences and Professions: Management, Education, and Law.

^6^The EFA was conducted using Principal Axis Factoring with Promax rotation and Eigenvalues >1.

^7^Each sub-factor corresponds to each of the scholarship dimensions or types: discovery, application, integration, teaching, and engagement. This is the same pattern for academic work focus and perceived institutional expectations.

^8^Braxton et al. (2002) developed a widely used faculty professional performance survey to assess the value given by the faculty to Boyer's 4 scholarship dimensions. However, they did not include scholarship of engagement as separate dimension. Their work combined with Boyer's (1990) original survey was used as the basis to develop our scholarship identity scale.

^9^There are several ways to compare two sets of preferences in MCDA. We chose this compatibility index because it is very easy to understand from a geometric point of view. However, while it is possible to visualize how close two vectors are with two- or three-dimensional coordinates, it is not possible to visualize it in our case where each vector has 5 coordinates; however, the mathematical principle is the same.

^10^This issue of consistently applying theoretical reasoning when covarying error terms is advocated by David Kenny: http://davidakenny.net/cm/respec.htm. He also recommends considering this action especially when the modification indices indicate that such an action would significantly reduce the chi-square. This second criterion was also true for this model.

^11^At the time of this writing, one major U.S. research institution has used our instrument for this purpose and a second one in Europe has started this process.
